# The effect of massage on the quality of life in patients recovering from COVID-19: A systematic review protocol

**DOI:** 10.1097/MD.0000000000020529

**Published:** 2020-06-05

**Authors:** Liu Wu, Yuting Dong, Jin Li, Ju Huang, Dengpeng Wen, Tao Peng, Jian Luo

**Affiliations:** a Department of Tuina, Hospital of Chengdu University of Traditional Chinese Medicine; b College of Acupuncture and Tuina, Chengdu University of Traditional Chinese Medicine, Sichuan, China.

**Keywords:** COVID-19, massage, systematic review

## Abstract

**Background:**

There is a worldwide outbreak of covid-19, and as the number of patients increases, more and more patients are recovering. Massage is used as an alternative therapy. Currently, there are no relevant articles for systematic review.

**Methods:**

We will search the randomized controlled trials related to acupuncture therapy and postoperative anorectal diseases from inception to January 2020. The following database is our focus area: the Cochrane Central Register of Controlled Trials (CENTRAL), PubMed, EMBASE, Web of Science, China National Knowledge Infrastructure, Chinese Biomedical Literature Database, and Wan-Fang Database. All published randomized controlled trials in English or Chinese related to massage for COVID-19 will be included. Primary outcome asthe influence of massage on the quality of life of convalescent patients. Secondary outcomes were accompanying symptoms (such as myalgia, expectoration, stuffiness, runny nose, pharyngalgia, anhelation, chest distress, dyspnea, crackles, headache, nausea, vomiting, anorexia, diarrhea) disappear rate, negative COVID-19 results rate on 2 consecutive occasions (not on the same day), average hospitalization time, clinical curative effect, and improved quality of life.

**Results:**

The results will provide a high-quality synthesis of current evidence for researchers in this subject area.

**Conclusion:**

The conclusion of our study will provide evidence to judge whether massage is an effective intervention on the quality of life in patients recovering.

**PROSPERO registration number:**

CRD42020181398

## 1 Introduction

In the end of 2019, a novel coronavirus, now known as SARS-CoV-2 (2019), suddenly emerged in Wuhan, China. The World Health Organization declared that the epidemic is a public health emergency of international concern on January 31, 2020. As of April 16, 2020, the emerging coronavirus infection, COVID-19, has been spreading worldwide, causing >2 million cases and >137 thousand of death.^[1]^ Coronoviruses have been reported as causes of mild and moderate respiratory infections for >50 years. Even though this group of viruses have been isolated from many different animals, bats are accepted major natural reservoir of coronaviruses.^[2,3]^

The coronavirus is a single-stranded RNA virus with a diameter of 80 ∼120 nm. It consists of 4 types, namely α-CoV, β-CoV, δ-CoV, and γ-CoV. Seven bats are the most important natural hosts. About 35% of the viruses they carry are coronaviruses, from which at least a dozen different coronaviruses have been identified so far.^[4]^ The COVID-19 was a novel class coronavirus with a circular or elliptic shape and a diameter of 60 to 140 nm.^[4,5]^ The typical coronavirus particle structure was observed under electron microscopy. Current studies have shown a 96.3% homology with the bat SARS-like coronavirus (BatCoV RaTG13), suggesting that bats may be the natural host for COVID-19.^[6]^ Coronaviruses (CoVs), mainly targeting human respiratory system, are responsible for health-threatening outbreaks including severe acute respiratory syndrome (SARS), Middle East respiratory syndrome, and lastly coronavirus disease 2019 (COVID-19).^[7]^ The main symptoms of the virus are a dry cough, fever and progressive dyspnea.^[8]^ Rapidly, this coronavirus, namely SARS-CoV-21, has spread worldwide, leading to a serious lung inflammation, acute respiratory distress syndrome, cardiac and renal injury, especially in patients with older age and comorbidities (diabetes mellitus, hypertension, and heart failure).^[9]^ The majority of cases (approximately 96%) occur with mild respiratory symptoms, some progress to pneumonia, acute respiratory distress syndrome, respiratory insufficiency, and multiorgan failure. The overall rate of deaths per number of diagnosed cases is 4.6%; ranging from 0.2% to 15% according to age group and other health problems.^[10]^

Although there are few reports on sequelae of COVID-19, the impact of the COVID-19 on people's quality of life is inevitable, especially in the population with sequelae of COVID-19. Complementary treatments, such as therapeutic massage (TM), are safe, noninvasive methods that hold promise for decreasing adverse side effects from cancer treatment.^[11]^ The National Center for Complementary and Integrative Health (NCCIH) generally employs the term complementary health approaches when discussing natural products and mind body practices used for various health conditions.^[12]^ Massage is a preventive and restorative therapy involving the systematic application of pressure to the skin, muscle, and connective tissue with the aim of improving blood and lymph circulation.^[13]^ Initially public emotional response to any pandemic is of extreme fear and uncertainty which usually drives toward negative societal behaviors and can involve public mental health concerns like anxiety, insomnia, depression aggression, frustration, and hysteria.^[14]^ Myers et al (2008) conducted a systematic review of TM in patients with cancer. Results demonstrated that TM enhanced comfort; decreased side effects of anxiety, pain, nausea, depression, and fatigue; and improved quality of life.^[15]^ Massage therapy is characterized by the excellent therapeutic effect on analgesia, no side effect, easy to operate, low economic burden, and more beneficial to promote patients to recovery.

Currently, there is still a lack of evidence-based medical evidence for the treatment of covid-19 in convalescent patients, and it is necessary for the improvement of mood management, anxiety, and quality of life in convalescent patients. Therefore, it is necessary to review it and provide evidence for clinicians

## 2 Methods

### 2.1 Study registration

The systematic review protocol has been registered in PROSPERO. The registration number is CRD42020181398; the consent of this protocol report is based on the Preferred Reporting Items for Systematic Reviews and Meta-Analyses Protocols statement guidelines.^[16]^

### 2.2 Inclusion criteria for study selection

#### 2.2.1 Type of study

We will include articles related to massage therapy of patients recovering from COVID-19. Due to language restrictions, we will search for articles in English and Chinese in order to get a more objective and true evaluation, all articles included are randomized controlled trial (RCT) type articles.

#### 2.2.2 Type of participant

All patients recovering from COVID-19 will be included regardless of sex, age, race, education, and economic status. Pregnant women, postoperative infections, psychopaths, and patients with severe cardiovascular and liver and kidney diseases will not be included.

#### 2.2.3 Type of intervention

Massage therapy including tuina and manipulation, whereas other traditional Chinese therapies such as acupuncture, moxibusition, cupping, and traditional Chinese medicine will be excluded. We will compare the following interventions: treatments other than massage (eg, usual or standard care, placebo, wait-list controls).

#### 2.2.4 Type of outcome measure

Primary outcom was the influence of massage on the quality of life of convalescent patients. Secondary outcomes were accompanying symptoms (such as myalgia, expectoration, stuffiness, runny nose, pharyngalgia, anhelation, chest distress, dyspnea, crackles, headache, nausea, vomiting, anorexia, diarrhea) disappear rate, negative COVID-19 results rate on 2 consecutive occasions (not on the same day), CT image improvement, average hospitalization time, occurrence rate of common type to severe form, clinical cure rate, and mortality.

### 2.3 Data sources

The following electronic databases will be searched from inception to April 2020: the Cochrane Central Register of Controlled Trials (CENTRAL), PubMed, EMBASE, Web of Science, China National Knowledge Infrastructure, Chinese Biomedical Literature Database, and Wan-Fang Database. About other sources, we also plan to manually search for the unpublished conference articles and the bibliography of established publications.

### 2.4 Search strategy

The search terms on PubMed are as follows: massage (eg, “acupoints” or “tuina” or “manipulation”); COVID-19 (eg, “Corona Virus Disease 2019” or “Corona Virus”); convalescence (eg, “rehabilitation” or “convalescent period” or “decubation”); randomized controlled trial (eg, “randomized” or “randomly” or “clinical trial”). Combinations of Medical Subject Headings (MeSH) and text words will be used. The same search term is used in electronic databases in China. These search terms are shown in Table 1.

**Table 1:**
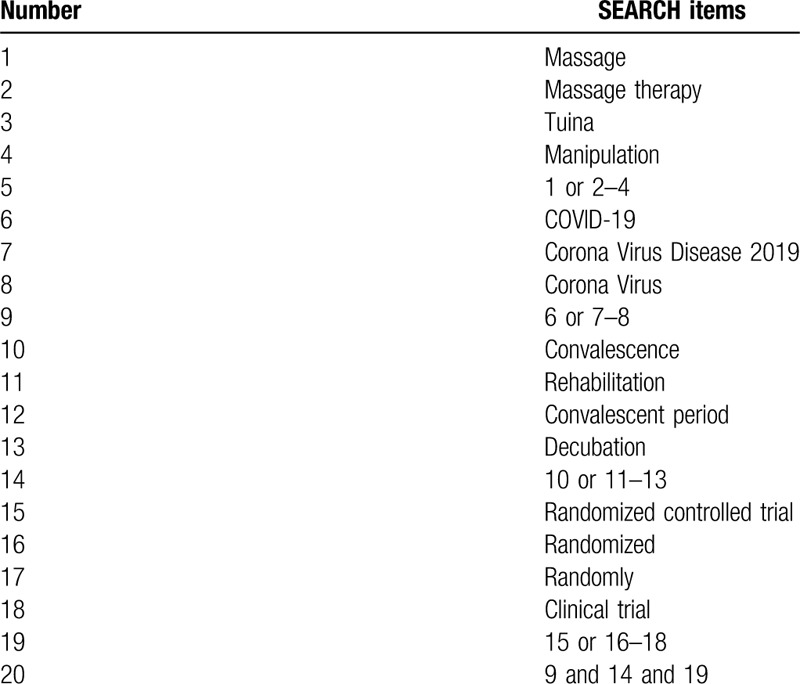
Search strategy for the PubMed database.

### 2.5 Data collection and analysis

#### 2.5.1 Selection of studies

**Figure 1:**
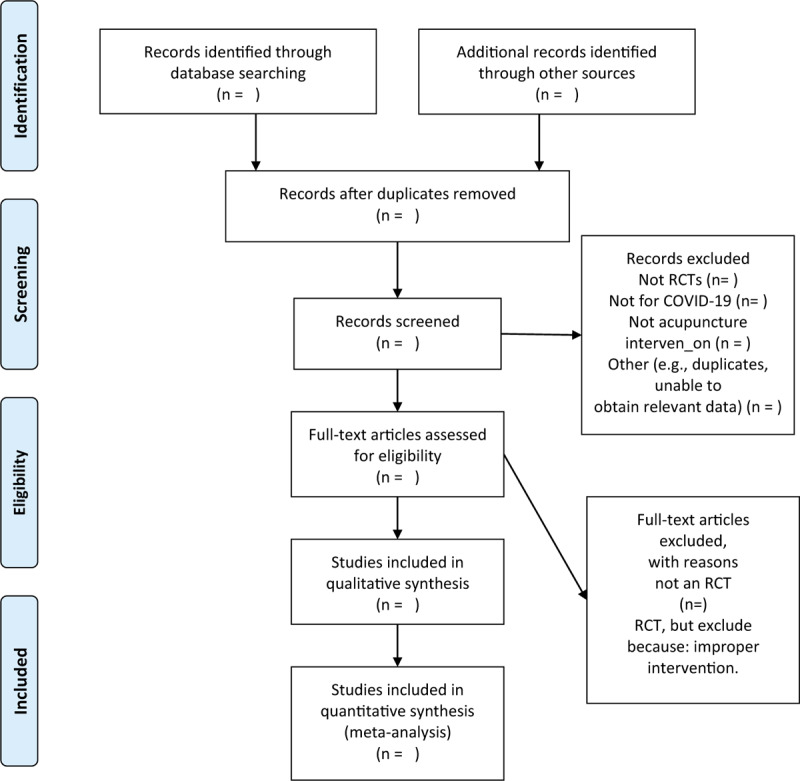
Flow chart of the study.

We chose the PRISMA flow chart to show the process of selecting literature for the entire study (Fig. 1). Before searching the literature, all reviewers will discuss and determine the screening criteria. After the screening requirements are clearly defined, the 2 reviewers (LW and YTD) will independently review and screen the literature. They screened the titles and abstracts of the literature, to get qualified studies, and then excluded some duplicate studies or studies with incomplete information. We will also try to obtain the full text, and the obtained literature will be managed by using EndNote software, V.X8 (United States). In case of disagreement between the 2 reviewers, discussions will be held with the third author (JL) for arbitration.

#### 2.5.2 Data extraction and management

The authors will strictly follow the inclusion criteria and select RCT articles related to the topic. Through the analysis of the article, we know participants’ characteristics (height, weight, sex), interventions, outcomes, the study characteristics (press, nationality, journals, research design), adverse reactions, among others. If there is any disagreement between the 2 authors in the literature data extraction, a third article participant (JH) will discuss the decision together. If there is a lack of data in the literature, we will contact the author or publisher as much as possible.

#### 2.5.3 Assessment of risk of bias in included studies

We will use the Cochrane collaborative tool to independently assess the risk of bias in the included studies. We will evaluate the following aspects of the article: sequence generation, assignment sequence hiding, blindness of participants and staff, outcome evaluators, incomplete result data, selective result reporting, and other sources of bias. The risk of bias is evaluated at 3 levels, namely, low risk, high risk, and ambiguity. If the information is vague, we will try to contact the author of the article.

#### 2.5.4 Measures of treatment effect

In this protocol, we will use 95% confidence interval (CI) risk ratio to rigorously analyze the dichotomous data. And for the continuous data, mean difference (MD) or standard MD is used to measure the efficacy of 95% CI.

#### 2.5.5 Unit of analysis issues

We will include data from parallel group design studies for meta-analysis. In these trials, we will collect and analyze individual measurements of each outcome for each participant.

#### 2.5.6 Management of missing data

We will try our best to ensure the integrity of the data. If the included RCT data are not complete, we will try every means to contact the corresponding author of the article, including sending emails or making a phone call. If the corresponding author cannot be contacted, we will remove the experiment with incomplete data. After data integrity is assured, intention analysis therapy and sensitivity analysis will be performed.

#### 2.5.7 Assessment of heterogeneity

For the detection of heterogeneity, the *I*^2^ test will be used to detect the heterogeneity among trials. When the *I*^2^ test value is <50% and *P* value >1, we think there is no heterogeneity between these trials, and when the *I*^2^ test value is >50% and the *P* value is <1, there is significant heterogeneity between these included trials. If significant differences are detected, we will analyze the possible causes of heterogeneity, and then we can use the random-effects model.

#### 2.5.8 Assessment of reporting biases

In this analysis, once >10 trials are included, funnel plots could be used to test for reporting bias.

#### 2.5.9 Data synthesis

We will use Review Manager Software (RevMan) V.5.3 (Copenhagen, Denmark) for data analysis and quantitative data synthesis. If there is no finding of statistical heterogeneity, the fixed-effect model is used for data synthesis. If there is significant statistical heterogeneity, we will use the random-effect model, and all participants will explore the possible causes from a clinical and methodological perspective and provide a descriptive or subgroup analysis.

#### 2.5.10 Subgroup analysis

Subgroup analysis will be performed to explain heterogeneity if possible. Factors such as different types of control interventions and different outcomes will be considered.

#### 2.5.11 Sensitivity analysis

Based on sample size, study design, heterogeneous quality, methodological quality, and statistical model, sensitivity analysis will be performed to exclude trials with quality defects and ensure the stability of the analysis results.

#### 2.5.12 Grading the quality of evidence

This article will use the evidence quality rating method to evaluate the results obtained from this analysis. GRADE is generally applied to a large amount of evidence. It has 4 evaluation levels, namely, high, medium, low, and very low. GRADE was used to evaluate the bias, inconsistencies, discontinuities, and inaccuracies of test results. In the context of the system review, quality reflects our confidence in the effectiveness of assessment.^[17]^

#### 2.5.13 Ethical review and informed consent of patients

Ethics and dissemination: The content of this article does not involve moral approval or ethical review and will be presented in print or at relevant conferences.

## 3 Discussion

With the increase in the number of diagnosed COVID-19, the number of cured COVID-19 is on the rise, and the number of people in the recovery period of COVID-19 is also on the rise. Patients with COVID-19 are often accompanied by a series of symptoms such as anxiety and insomnia. There is evidence that supports the effectiveness and safety of massage for anxiety, insomnia, and aches and pains all over the body.^[15]^

This review is divided into 4 parts: identification, literature inclusion, data extraction, and data synthesis. It will systematically review the RCT literature; this review will evaluate the effectiveness of acupuncture in treating COVID-19 convalescent patients. There are also limitations in our research and the language bias here is that we only search for Chinese and English documents. Our study may provide a basis for clinicians to choose replacement therapy for further study in the future.

## Author contributions

**Conceptualization:** Liu Wu.

**Data curation:** Jin Li, Ju Huang, Dengpeng Wen, Tao Peng.

**Formal analysis:** Ju Huang, Dengpeng Wen, Tao Peng.

**Funding acquisition:** Jian Luo.

**Resources:** Jian Luo.

**Software:** Yuting Dong.

**Writing – original draft:** Liu Wu.

**Writing – review & editing:** Yuting Dong, Jin Li, Jian Luo.
